# The evaluation of non-anesthetic computed tomography for detection of pulmonary parenchyma in feline mammary gland carcinoma: a preliminary study

**DOI:** 10.1186/s12917-021-02950-6

**Published:** 2021-07-06

**Authors:** Auraiwan Klaengkaew, Somchin Sutthigran, Ninlawan Thammasiri, Kittiporn Yuwatanakorn, Chutimon Thanaboonnipat, Suppawiwat Ponglowhapan, Nan Choisunirachon

**Affiliations:** 1 Department of Surgery, Faculty of Veterinary Science, Chulalongkorn University, 39 Henri-Dunant Road, Wangmai, Pathumwan, 10330 Bangkok, Thailand; 2 Department of Obstetrics, Gynaecology and Reproduction, Research Unit of Obstetrics and Reproduction in Animals, Faculty of Veterinary Science, Chulalongkorn University, 10330 Bangkok, Thailand

**Keywords:** Atelectasis, Cat, Computed tomography, Lung, Mammary gland carcinoma, Radiography

## Abstract

**Background:**

Thoracic radiography in awake cats is a common procedure for the evaluation of pulmonary metastasis in feline mammary gland carcinoma (MGC). However, due to poor sensitivity, computed tomography (CT) is progressively taking its place. To perform CT in animals, general anesthesia is normally preferred but can cause lung atelectasis, affecting lung interpretation. Besides, MGC is often found in senile cats that are concurrently affected with other diseases, increasing anesthetic risk. Therefore, this study was aimed at comparing the effect of anesthesia on lung atelectasis observed through CT in clinically healthy cats and comparing the feasibility of non-anesthetic CT with non-anesthetic radiography in the detection of lung lesions in feline MGC. Thoracic CTs from anesthetized, clinically healthy cats and non-anesthetized either clinically healthy cats or MGC-affected cats were reviewed. In clinically healthy cats, motion artifacts and characteristics of lung atelectasis were observed and compared. In MGC-affected cats, motion artifacts were observed and compared to clinically healthy cats, and the number of MGC-affected cats, the number and characteristics of lung lesions were compared between non-anesthetic thoracic CT and radiography.

**Results:**

Anesthesia significantly increased lung CT attenuation (*P* = 0.0047) and was significantly correlated with lung atelectasis (OR = 15; CI 2.02–111.18; *P* = 0.0081), particularly of the cranial lung lobe. Nonetheless, significantly higher motion artifacts in the caudal thoracic area were found in non-anesthetized healthy cats (*P* = 0.0146), but comparable low motion artifacts were observed in anesthetized healthy and MGC-affected cats. Non-anesthetic CT revealed higher numbers of MGC-affected cats and pulmonary nodules with a significantly lower nodular diameter (*P* = 0.0041) than those observed on radiographs. The smallest nodular diameters detected on radiographs and CT were 2.5 and 1.0 mm, respectively. Furthermore, CT showed additional information such as intra-thoracic lymphadenopathy, that could not be seen on radiographs.

**Conclusions:**

Despite the motion artifacts, CT without anesthesia is a sensitive technique as it provides better lung inflation. Furthermore, compared to non-anesthetic radiography, non-anesthetic CT provided more information such as higher number of pulmonary nodules of a smaller size, including more distinct intra-thoracic lesions, such as lymphadenopathy, in MGC-affected cats.

## Background

Mammary gland carcinoma (MGC) is the third most frequent feline neoplasms [1, 2]. It has been reported that MGC usually occurs in senile, female cats with an age ranging between 10 and 14 years [3, 4]. Due to aggressive biological behaviors, feline MGC normally demonstrates rapid growth and commonly metastasizes to regional lymph nodes and lungs [5, 6]. The survival period of feline MGC has been reported as being less than 6–12 months [5, 6]. Therefore, early diagnosis and accurate clinical staging observed through metastatic condition are essential for therapeutic planning and prognosis of feline MGC [1].

Diagnostic imaging is a non-invasive method for identification of MGC metastases. Radiography is an initial procedure for lung metastatic screening. Despite being a cheap, low radiation and generally applicable option, it has been reported in dogs that radiographs showed less sensitivity in detecting lung lesions both of size and number [7, 8], However, to our knowledge, this information has not yet been explored in cats. Computed tomography (CT) is currently described as a superior modality in detecting lung lesions in dogs and cats [8–10]. CT has a higher ability to detect a smaller nodule without superimposition than radiographs in dogs [8]. To perform CT in animals, general anesthesia is usually required. However, general anesthesia can be a risk for respiratory compromised, feline patients [11]. Since feline MGC is commonly found in senile cats. These cats may be concurrently affected with other diseases increasing anesthetic risk such as cardiac or renal diseases. In addition, it has been suggested that anesthesia could prolong the duration of the CT procedure and it more importantly, caused lung atelectasis as seen in humans and cats [9, 12, 13].

As there is no information on the use of pulmonary CT, especially non-anesthetic CT for detecting lung metastases in feline MGC. Therefore, the objectives of this study were: (i) to evaluate the effect of anesthesia on the pulmonary appearance of healthy cats by means of the motion artifact and lung characteristic; and (ii) to compare the diagnostic feasibility between non-anesthetic CT and non-anesthetic radiographs for detecting lung lesions in MGC-affected cats. The hypotheses were that anesthesia can influence the appearance of lung parenchyma of healthy cats by means of the motion artifact and lung atelectasis, and non-anesthetic CT is superior to non-anesthetic radiographs for detecting MGC metastatic lung lesions in terms of number, location and distinctness.

## Results

### Clinical demographic data

The number of attending cats in this study were 12, 12 and 20 cats for group (Gr.) 1 (the anesthetized healthy cats), Gr. 2 (the non-anesthetized healthy cats) and Gr. 3 (MGC-affected cats), respectively. All information concerning cats in each group are reported in Table 1. Gr. 1 and Gr. 2 had a significantly lower age than Gr. 3. (*P* < 0.0001 and *P* = 0.017, respectively) but the age between Gr.1 and Gr.2 and the body weight (BW) among groups were comparable (*P* = 0.2522 for age between Gr.1 and Gr. 2 and *P* = 0.7434 for BW among groups).

**Table 1:** Clinical demographic data of clinically healthy cats, which divided into the anesthetized healthy cats (Gr. 1) and non-anesthetized healthy cats (Gr. 2), and mammary gland carcinoma affected cats (Gr. 3)

Parameters			Gr. 1	Gr. 2	Gr.3
Number			12	12	20
Age (months)			19.3 ± 23.6^α^ (7.0–84.0)	41.0 ± 10.8^α^ (24.0–60.0)	131.2 ± 41.0^α^ (24.0-216.0)
Body weight (kg)			3.4 ± 0.9^β^ (1.5–4.7)	3.6 ± 0.8 ^β^ (2.0–5.0)	3.7 ± 0.8 ^β^ (2.2-5.0)
Sex*					
	Male		5	6	-
		Intact	3	-	-
		Castrated	2	6	-
	Female		7	6	20
		Intact	3	-	5
		Spayed	4	6	15*
Breed					
	Domestic short hair		9	7	16
	Mixed breed		2	2	-
	American short hair		-	2	-
	British short hair		-	1	-
	Scottish fold		1	-	-
	Persian		-	-	4

* Sex and gonadal status of the cat at the experiment date

^α^Age was compared among groups using Kruskal-Wallis test: *P* < 0.0001.

^α^Age was compared between groups using Dunn’s Multiple comparisons test:

Gr. 1 vs. Gr. 2: *P* = 0.2522.

Gr. 1 vs. Gr. 3: *P* < 0.0001.

Gr. 2 vs. Gr.3: *P* = 0.0017.

^β^Body weight was compared among group using one-way ANOVA: *P* = 0.7434

### Motion artifacts on computed tomographic images

The number of cats and degree of motion artifacts observed on CT among groups are shown in Table 2. In Gr. 1 and Gr. 2, motion artifacts were detected at the caudal area of the thoracic cavity. In Gr. 3, nine cats revealed motion artifacts at the caudal thoracic area whereas one cat showed a motion artifact at the cranial part. Gr.1 and Gr. 3 had significantly less motion artifacts than Gr. 2 (*P* = 0.0146 and *P* = 0.0246, respectively) but motion artifacts between Gr. 1 and Gr. 3 did not differ significantly (*P* > 0.9999).

**Table 2:** The motion artifact among anesthetized healthy cats (Gr. 1), non-anesthetized healthy cats (Gr. 2) and mammary gland adenocarcinoma affected cats (Gr. 3) on thoracic computed tomography

Group	Number of cats			Average motion artifact score
	**Grade 0**	**Grade 1**	**Grade 2**	
Gr. 1	8/12 (66.7 %)	3/12 (25.0 %)	1/12 (8.3 %)	0.4 ± 0.6
Gr.2	2/12 (16.6 %)	5/12 (41.7 %)	5/12 (41.7 %)	1.2 ± 0.7
Gr. 3	10/20 (50.0 %)	10/20 (50.0 %)	0/20 (0 %)	0.5 ± 0.5

Motion artifacts were compared among groups using Kruskal-Wallis test: *P* = 0.0083

Motion artifacts were compared between groups using Dunn’s Multiple comparisons test:

Gr. 1 vs. Gr. 2: *P* = 0.0146.

Gr. 1 vs. Gr. 3: *P* > 0.9999.

Gr. 2 vs. Gr.3: *P* = 0.0246

### Lung atelectasis of healthy cats on computed tomographic images

Information on lung atelectasis such as the number of cats, number of lungs, average attenuation number and characteristics among normal lung tissue (NL), poorly lung aeration (PA) and non-lung aeration (NA) clinically healthy cats in Gr. 1 and Gr. 2 were reported and are compared in Table 3. At NL, there was no difference in lung attenuation between groups (*P* = 0.1089). Whereas at PA or NA, Gr.1 had a significantly higher attenuation number than that of the Gr. 2 (*P* = 0.0047). Considering the location, the most frequent locations of lung atelectasis were found at the right cranial lung (RtCr), the right middle lung (RtMd) and the cranial part of left cranial lung (LtCrCr) lobes, especially at the ventral area, and the peri-bronchial area was the common intra-parenchymal location (Table 4). There was a significant association between anesthetic procedure and the presence of lung atelectasis (OR = 15; CI 2.02–111.18; *P* = 0.0081).

**Table 3:** The number of cats, the number of lungs, average attenuation number and characteristics of atelectatic lung (HU) in anesthetized healthy cats (Gr. 1) and non-anesthetized healthy cats (Gr. 2)

Parameters		Gr. 1*	Gr.2*
Number of atelectatic cats		10/12 (83.3 %)	3/12 (25.0 %)
Normal lung			
	Number of lung lobes	60/84 (71.4 %)	80/84 (95.2 %)
	Attenuation number (HU)	-698.4 ± 71.9 (-678.1 to -721.2)	-666.3 ± 56.0 (-554.6 to -731.1)
Atelectatic lung			
	Total number of lung lobes	24/84^α^ (28.5 %)	4/84^α^ (4.7 %)
	Total attenuation number (HU)	-126.0 ± 70.2 (-39.7 to -364.3)	-381.5 ± 135.8 (-215.4 to -426.9)
	-Poorly aerated lung tissue	22/84 (26.2 %)	4/84 (4.7 %)
	Attenuation number (HU)	-174.0 ± 27.8 (-120.7 to -364.3)	-381.5 ± 135.8 (-215.4 to -426.9)
	-Non-aerated lung	2/84 (2.3 %)	0/84 (0 %)
	Attenuation number (HU)	-49.5 ± 13.8 (-39.7 to -59.3)	0
	Characteristics -Peripheral Number of lung lobes -Diffused Number of lung lobes -Peribronchial Number of lung lobes	7/24 (29.2 %) 1/24 (4.2 %) 16/24 (66.6 %)	1/4 (25.0 %) 3/4 (75.0 %) 0/4 (0 %)
	Locations		
	-Dorsal	6/24 (25.0 %)	1/4 (25.0 %)
	-Ventral	17/24 (70.8 %)	0/4 (0.0 %)
	-Diffused	1/24 (4.2 %)	3/4 (75.0 %)

*The total number of cats and total number of lung lobe in each group were 12 and 84, respectively

^α^The association between anesthetic procedure and the presence of lung atelectasis was evaluated using the odds ratio: (OR = 15; CI 2.02–111.18; *P* = 0.0081)

**Table 4:** Distribution of poor pulmonary inflation in all affected cats (*n* = 13 cats) and each group of anesthetized healthy cats (Gr. 1) and non-anesthetized healthy cats (Gr. 2)

Lung atelectasis	Locations						
	**RtCr**	**RtMd**	**RtAcc**	**RtCa**	**LtCrCr**	**LtCrCa**	**LtCa**
Total affected cats	9	7	0	4	2	6	0
Poorly-aerated lung	7	7	0	4	2	6	0
-Gr.1	5	7	0	4	1	5	0
-Gr.2	2	0	0	0	1	1	0
Non-aerated lung	2	0	0	0	0	0	0
-Gr.1	2	0	0	0	0	0	0
-Gr.2	0	0	0	0	0	0	0

### Comparison of feline mammary gland carcinoma and pulmonary lesions between on non-anesthetic thoracic radiograph and on computed tomography

**Fig. 1:**
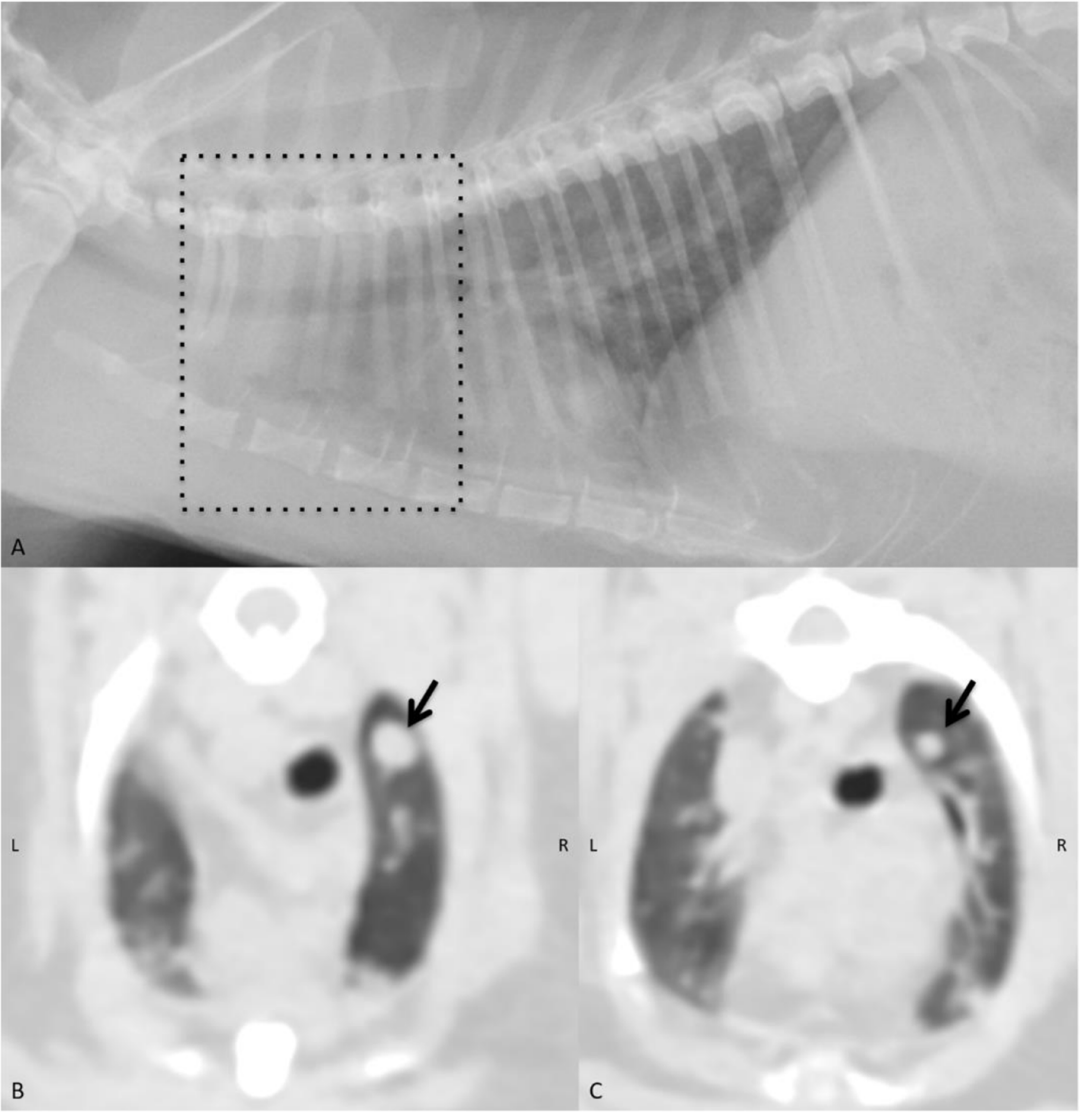
A left lateral thoracic radiograph (**A**) and transverse computed tomographic image (**B** and **C**) of a mammary gland carcinoma affected cat. On radiograph (A), non-distinct ill-defined pulmonary nodules were found throughout the right inflated lung lobe. However, on transverse computed tomographic image (**B **and **C**), all pulmonary nodules, especially at the cranial thoracic compartment as seen on the thoracic radiograph (dash box, **A**) revealed clearly nodal margination (arrows)

**Fig. 2:**
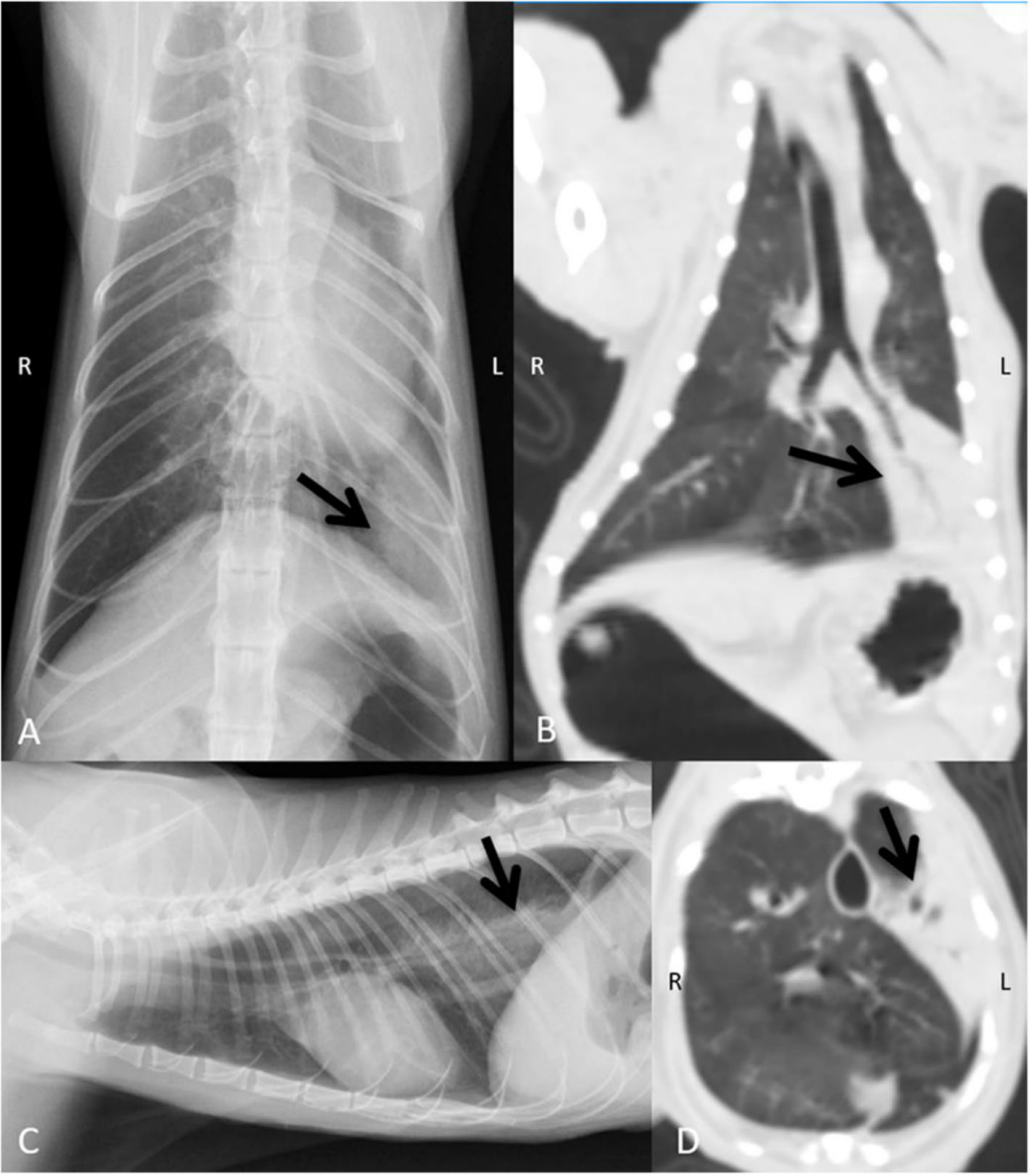
Ventrodorsal (**A**) and right lateral (**C**) thoracic radiographs and dorsal (**B**) and transverse (**D**) computed tomographic images of a mammary gland carcinoma affected cat. On thoracic radiograph (**A **and **C**), a focal, patchy lung consolidation was found at the left caudal lung lobe (arrows). However, on transverse computed tomographic images (**B** and **D**), the location of affected lung parenchyma was revealed as the lobar atelectatic area (arrows)

The radiographic and CT lung information such as number of cats, lesion distribution, lesion pattern, number of nodules, location, and size are reported in Table 5. Despite poor margination of detected nodules, CT unveiled images of nodule distinctness clearer than those observed on radiographs (Fig. 1 A-C). Lung nodules could not be detected on radiographs in three cats and those nodules were smaller than 2.5 mm. Radiographically, there was one cat affected by the left caudal lung lobe (LtCa) consolidation without a pulmonary nodule (Fig. 2 A and 2 C). However, on CT, this cat was later noted as having pulmonary nodules and the previous lung consolidation was noted as having moderately decreased lung volume with an alveolar pattern that was suspected to be atelectatic area (Fig. 2B and D). CT can significantly detect lesser diameter pulmonary nodules than thoracic radiographs (*P* = 0.0041). In addition, five cats showed sternal lymphadenopathy only on CT, but not on radiographs.

**Table 5:** The radiographic and computed tomographic lung information such as the number of cats, lesion distribution, lesion pattern, the number of nodules, location, and size in twenty mammary gland adenocarcinoma affected cats

Lung metastasis		Radiograph	Computed tomography
Number of cats		7/20 (35.0 %)	10/20 (50.0 %)
Distribution of nodules			
	Focal lesion	4/7 (57.1 %)	3/10 (30.0 %)
	Diffuse lesion	3/7 (42.9 %)	7/10 (70.0 %)
Lesional pattern			
	Ill-defined nodules	7/20 (35.0 %)	10/20 (50.0 %)
	Lung consolidation without a nodule	1/20 (5.0 %)	0/20 (0 %)
Number of nodules		16	65
Location of nodules			
	Right cranial compartment	2/16 (12.5 %)	11/65 (16.9 %)
	Right middle compartment	5/16 (31.2 %)	8/65 (12.3 %)
	Right caudal compartment	3/16 (18.7 %)	20/65 (30.7 %)
	Left cranial compartment	0/16 (0 %)	7/65 (10.7 %)
	Left middle compartment	0/16 (0 %)	6/65 (9.2 %)
	Left caudal compartment	6/16 (37.5 %)	13/65 (20.0 %)
Size of nodules (mm)		5.8 ± 2.2 (2.5–10.2)	3.8 ± 1.9 (1.0-8.4)

## Discussion

The precise evaluation of clinical staging is an important factor to indicate the proper therapeutic methods and precise prognosis in several malignancies including feline MGC [4, 6]. Although several studies in dogs and cats have reported that CT was more precise and provided additional diagnostic information of thorax compared to thoracic radiography [7–9], general anesthesia during the CT procedure can be one of the life-threatening risks to senile, MGC-affected cats that may have other underlying impairments such as renal or cardiac diseases. Therefore, non-anesthetic CT for evaluation of pulmonary nodules could provide more safer and sensitive information for MGC-affected cats.

Anesthesia can cause lung atelectasis which interferes with the precision of lung evaluation in feline patients [9]. Characteristics of lung atelectasis in animals can include an interstitial or alveolar pattern [14]. Both patterns can mislead the diagnosis of pathologic lung lesions. A previous study in humans indicated that lung atelectasis appears on CT within five minutes following induction of anesthesia [15]. Similarly, in dogs, lung attenuation on CT was altered within three to eight minutes of anesthesia [16, 17]. In addition, it has been reported in human medicine that age and BW influence lung volume and oxygenation impairment. These impairments are higher with increased age or body mass index [18, 19]. However, the effect of these factors on lung volume and oxygen impairment in cats has never been documented. Due to the non-significant differences of age and BW between clinically healthy cats and lung atelectasis in this study, the evidence of lung atelectasis in clinically healthy cats might be influenced by anesthetic procedure. Nevertheless, further studies that evaluate an effect of age or BW on lung volume in healthy cats under a control anesthetic protocol would provide additional information.

Motion artifacts were higher in non-anesthetized cats that had a normal respiratory rate of 20–30 breaths/min compared to anesthetized cats that maintained the anesthetic condition using a control ventilator at 12 breaths/min. The prolongation of respiration by a ventilator comparing to that of the cats with consciousness could eliminate motion artifacts. Despite a higher respiratory rate, non-anesthetized cats revealed a moderate motion artifact score that may affect diagnostic quality [20], especially at the caudal lung lobes suggesting the motion of the diaphragm [9].

In this study, anesthetized cats had significantly higher lung atelectasis than non-anesthetized cats in line with previous studies in dogs [21]. Lung atelectasis in cat may be caused by lung compression similar to human report [22]. Moreover, inhalation anesthesia was suggested in human medicine as being one of the factors inducing atelectasis by the reduction of functional residual capacity due to the impairment of intercostal muscle function and the reduction of thoracic wall traction [22]. Besides, the lack of spontaneous deep inspiration phase resulting in the decrease of lung surfactant has also been noted [23]. The cranial displacement of the diaphragm by the increasing abdominal pressure during anesthesia in humans was reported to may increase the risk of atelectasis due to the decreasing thoracic cavity volume [24]. In addition to the anatomical factors during anesthesia, the administration of 100 % oxygen for carrying anesthetic gas may also cause lung atelectasis. Previous studies in dogs and cats reported that the higher oxygen concentration during general anesthesia was associated with a significantly increased degree of lung atelectasis [10, 21, 25, 26]. It has been reported that the 100 % oxygen for carrying anesthetic gas during the inhalation anesthesia induced more lung atelectatic formation in cats than those who received the 40 % oxygen [10, 26].

The main atelectatic location in a previous feline study was reported as being distributed at a caudal lung lobe, close to the diaphragm [26]. However, the most frequent atelectatic locations in this study were found at the ventral portions of the RtCr, RtMd, and LtCrCr which are almost similar to another feline report [27]. The discrepancy among studies may be due to the compression force from the abdominal organs associated with the use of muscle relaxants during general anesthesia [26], whereas the atelectasis in the present study may be due to a smaller lung volume compared to other areas. A reduction of ventilation in the small lung lobes of cats can cause atelectatic development during general anesthesia similar to dogs [28]. Many atelectatic lungs revealed a peri-bronchial pattern at the ventral portion of the lung lobe. Therefore, utilization of CT as a diagnostic modality for pulmonary diseases, especially the bronchial area in cats without non-anesthetic methods or without the breath-hold, general anesthesia technique, should be avoided to prevent misinterpretation due to pulmonary atelectasis.

Non-anesthetic, thoracic radiographs and CT were done in MGC-affected cats. Both results can be comparable by means of the status of pulmonary inflation; the positionings for evaluation were different though. Thoracic CT can identify a greater number of lung metastatic affected cats and a higher number of pulmonary nodules of a smaller size compared with thoracic radiographs. Due to the transverse display, CT can reduce the superimposition that provides the greatest impact on assessment of small pulmonary nodules similar to dogs [7]. Ill-defined nodules or diffuse pulmonary patterns observed on thoracic radiography have been reported in 83 % of cats suffering from MGC [29]. However, CT showed a clearer border of those lung nodules as observed in our study. Therefore, the sensitive diagnostic method of CT helps assess the prognostic survival period. It is well known that therapeutic treatment for animals presenting with metastatic lesions is a candidate for poor prognosis and that standard chemotherapy is commonly ineffective [30].

The most frequent locations of lung nodules occurred at the caudal thoracic compartment. Although this area was prone to being affected with motion artifacts in non-anesthetized healthy cats, this motion artifact was unlikely to be seen in the MGC-affected cats. The less motion artifacts at this area could be related to advancing age and health conditions of MGC-affected cats. They were senile and in an unhealthy condition that may cause a lower respiratory rate compared to young healthy cats, the actual respiratory rates between groups of cats during the CT scan was difficult to detect though. Thus, the utilization of CT without general anesthesia could be superior for evaluating lung metastatic nodules in feline MGC. Moreover, CT provided additional information involving metastatic processes such as sternal lymphadenopathy and pleural effusion, which were more difficult to detect on thoracic radiograph in the case of mild alterations.

Regarding clinical situations in limited circumstances and the regulation of animal use policy, we were unable to perform cytological/histological examination of lung nodules in all feline MGCs. It is possible that some pulmonary nodules were granulomas or lung fibrosis. In addition, in clinical situations, it was difficult to set up an age-match control study to evaluate the lung condition because senile cats are prone to be affected with other diseases related to anesthetic risk. Moreover, in MGC, the comparisons between clinical stages and tissue subtypes could not be done due to the insufficient number of samples. Therefore, the comparison of the diagnostic evidence with histopathology and/or clinical staging warrants further investigation.

## Conclusions

General anesthesia clearly resulted in lung atelectasis, particularly of the cranial lung lobe whereas non-anesthetic pulmonary parenchyma on CT showed caudal thoracic motion artifacts. On the other hand, in senile MGC-affected cats, non-anesthetic CT was recommended because it provided less motion artifact and high sensitivity to detecting very small-sized lung nodules including intra-thoracic lymphadenopathy and pleural effusion more clearly than that observed on conventional, non-anesthetic radiographs.

## Methods

### Animals

This study was divided into two parts: an investigation of the anesthetic effect on lung characteristics in clinically healthy cats and a comparison of diagnostic accuracy between non-anesthetic CT and conventional, non-anesthetic thoracic radiographs for detecting MGC lung lesions. All cats conducting experiments were presented to The Small Animal Hospital, Faculty of Veterinary Science, Chulalongkorn University from January 2017 to February 2020. The study was approved by The Chulalongkorn University Animal Care and Use Committee (CU-IACUC); the approval numbers 1631073, 1831094 and 1931039 for cats in Gr. 1, 2 and 3, respectively. All experiments were carried out in accordance with relevant guidelines and regulations. All owners of the cats were informed consents and then all consents were written by the the cat owners.

Cats were divided into three groups: group 1 (Gr. 1: the anesthetized healthy cats); group 2 (Gr. 2: the non-anesthetized healthy cats); and group 3 (Gr. 3: MGC-affected cats). The inclusion criteria for cats in Gr. 1 and Gr. 2 were clinically healthy cats showing unremarkable results through history taking, physical examinations, thoracic auscultation, hematology, and basic serum biochemistry, thoracic radiographs and abdominal ultrasound. Cats that revealed any abnormalities from those health screenings were excluded. Gr. 3 were MGC-affected cats that had previous cytologic or histopathologic confirmation of mammary gland adenocarcinoma. Cats that were pregnant, were in lactating period, and had a history or clinical signs of other respiratory such as asthma or previous pneumonia or other intra-thoracic abnormalities unrelated to MGC, e.g. mediastinal abnormality, cardiomegaly, or pleural effusion, were excluded from this study. Clinical demographic information was recorded.

### Radiography for MGC-affected cats

Non-anesthetic, thoracic radiographs were performed using the standard institutional procedure. Right lateral, left lateral, and ventrodorsal thoracic radiographs were obtained. In Gr. 3, thoracic radiographs were primarily noted as either positive or negative for lung nodules, enlargement of intrathoracic lymph nodes and evidence of pleural effusion. Subsequently, each lateral radiograph was divided into three compartments of cranial (thoracic inlet to cranial cardiac silhouette), middle (cranial cardiac silhouette to cardiac apex), and caudal parts (cardiac apex to caudodorsal lung area). The number, size, and contour of lung nodules on each view and each area were then recorded. Furthermore, interlobular fissures were observed on the ventrodorsal radiographs for pleural effusion.

### Computed tomography

Prior to CT, cats in Gr.1 were sedated using acepromazine maleate (0.03 mg/kg, Combistress, Pharmaceuticals ND) and tramadol hydrochloride (2 mg/kg, Tramache, Harson Laboratories) intramuscularly, followed by induction with propofol (2–4 mg/kg, Propofol-Lipuro 1 % Ampoule, B. Braun) intravenously. After endotracheal intubation, anesthesia was maintained with 2–2.5 % of isoflurane in 100 % oxygen using a pressure controlled-ventilator (SV-2000, SORA MEDICAL TECH) at a rate of 12 breaths/minute, tidal volume at 15 ml/kg and maximal intratracheal pressure at 15 cmH2O without the breath-hold technique. Pre-contrast enhanced CT was obtained at approximately 10 min after intubation. On the other hand, cats in Gr. 2 and Gr. 3 were manually restrained in a plastic carrying box and supported by rolled towels for the desired position. As soon as positioning was achieved, CT images were obtained using a 64-slice, multi-detector CT scanner (Optima 660, GE Healthcare). During CT acquisition, all cats were in sternal recumbency with the head pointed into the CT gantry and the thoracic cavity perpendicular to the isocenter of the CT scan planes. Non-contrast enhanced CT was acquired at 120 kilovoltage peak (kVp), automate milliamperage (mA), effective slice thickness at 0.625 mm, collimator pitch at 0.935 mm, matrix size of 512 × 512 (isotropic voxels) and the field of view (FOV) was set to cover the whole area of the cats or the positioning device. CT images were collected in the Digital Imaging and Communications in Medicine (DICOM) format and analyzed with DICOM image viewer software (Osirix®, Pixmeo SARL).

A pulmonary window at 1400 Hounsfield unit (HU) of window width (WW) and − 500 HU of window level (WL) was applied to transverse CT images to evaluate each hemithorax. At first, motion artifacts were evaluated. Motion artifacts were classified into three grades: grade 0 (G0): an absence of image blurring due to respiratory motion; grade 1 (G1): the mild to moderate presence of image blurring that did not influence diagnostic quality; grade 2 (G2): severe presence of image blurring causing poor diagnostic quality [10]. Subsequently, the lung parenchyma was observed for lung atelectasis. The attenuation number at each of right cranial lung lobe (RtCr), right middle lung lobe (RtMd), right caudal lung lobe (RtCa), right accessory lung lobe (RtAcc), cranial part of left cranial lung lobe (LtCrCr), caudal part of left cranial lung lobe (LtCrCa), and left caudal lung lobe (LtCa) was observed by drawing a 5 mm^2^ of region of interest (ROI) and comparing them. The ROI was selectively drawn on the lung parenchyma without any distinct pulmonary vessels or airways. Lung was defined as normal lung tissue (NL) if lung attenuation was between − 900 and − 501 HU; poorly lung aeration (PA) if lung attenuation was between − 500 and − 101 HU; and non-lung aeration (NA) if lung attenuation was between − 100 and 100 HU [20]. Lung atelectasis was noted according to anatomical lobes and intra-parenchymal locations such as peripheral, peribronchial, or diffused patterns. Furthermore, the distribution of lung atelectasis was classified as being in dorsal, ventral or diffuse lung areas.

In Gr. 3, the number, diameters, and contours of lung nodules were observed on the transverse CT image and information was recorded for each area similar to the thoracic radiographs. Intra-thoracic lymphadenopathy and signs of pleural effusion were observed and noted.

### Statistical analysis

Descriptive statistics were used to present quantitative clinical demographic data. All statistical comparisons were conducted using Prism7 (GraphPad Software, San Diego, CA, USA). The normality test for each data set was primarily determined by the Shapiro-Wilk test. Age, BW, and motion artifacts among groups were compared using the Kruskal-Wallis test with Dunn’s multiple comparisons test or one-way ANOVA. The attenuation number of lungs between groups of clinically healthy cats was compared using an unpaired t-test whereas the association between anesthetic procedure and the presence of lung atelectasis was evaluated using the odds ratio. In addition, the number of lung nodules including size detected on CT and thoracic radiographs were compared using a Wilcoxon test. *P* < 0.05 was considered statistically significant.

## Data Availability

The datasets used and/or analyzed during the current study are available from the corresponding author on reasonable request.

## Abbreviations

BW: Body weight
CI: Confidence interval
CT: Computed tomography
CU-ACUC: Chulalongkorn University Animal Care and Use Committee
DICOM: Digital Imaging and Communications in Medicine
FOV: Field of view
Group: Gr
HU: Hounsfield unit
kg: Kilogram
kVp: Kilovoltage peak
LtCa: Left caudal lung lobe
LtCrCa: Caudal part of left cranial lung lobe
LtCrCr: Cranial part of left cranial lung lobe
mA: Milliamperage
mg: Milligram
MGC: Mammary gland carcinoma
mm: Millimeter
NA: Non-lung aeration
NL: Normal lung tissue
OR: Odd ratio
PA: Poorly lung aeration
ROI: Region of interest
RtAcc: Right accessory lung lobe
RtCa: Right caudal lung lobe
RtCr: Right cranial lung lobe
RtMD: Right middle lung lobe
WL: Window level
WW: Window width
